# What I have changed my mind about and why: public health and technology perspectives in the field of trauma studies

**DOI:** 10.1080/20008198.2017.1372007

**Published:** 2017-10-27

**Authors:** Paula P. Schnurr, Richard Bryant, Lucy Berliner, Dean G. Kilpatrick, Albert Rizzo, Josef I. Ruzek

**Affiliations:** ^a^ National Center for PTSD, White River Junction, VT, USA; ^b^ Geisel School of Medicine, Hanover, NH, USA; ^c^ University of New South Wales, Sydney, Australia; ^d^ Harborview Center for Sexual Assault and Traumatic Stress, Seattle, WA, USA; ^e^ Medical University of South Carolina, Charleston, SC, USA; ^f^ University of Southern California, Los Angeles, CA, USA; ^g^ Stanford University School of Medicine, Palo Alto, CA, USA

**Keywords:** Public health, technology, treatment effectiveness, dissemination, implementation, virtual reality

## Abstract

**Background**: This paper is based on a panel discussion at the 32nd annual meeting of the International Society for Traumatic Stress Studies in Dallas, Texas, in November 2016.

**Objective**: Paula Schnurr convened a panel of experts in the fields of public health and technology to address the topic: ‘What I have changed my mind about and why.’

**Method**: The panel included Richard Bryant, Lucy Berliner, Dean Kilpatrick, Albert (‘Skip’) Rizzo, and Josef Ruzek.

**Results**: Panellists discussed innovative strategies for the dissemination of scientific knowledge and evidence-based treatment.

**Conclusions**: Although there are effective treatments, there is a need to enhance the effectiveness of these treatments. There also is a need to develop simpler, low-cost strategies to disseminate effective treatments. However, technology approaches also offer pathways to increased dissemination. Researchers must communicate scientific findings more effectively to impact public opinion and public policy.

## Abstract (es)

**Planteamiento**: Este documento se basa en un debate en la 32ª reunión anual de la Sociedad Internacional para el Estudio del Estrés Traumático en Dallas, Texas, en noviembre de 2016.

**Objetivo**: Paula Schnurr convocó a un panel de expertos en las áreas de salud pública y tecnología para abordar el tema: ‘Sobre qué he cambiado de opinión y por qué’.

**Método**: El panel incluyó a Richard Bryant, Lucy Berliner, Dean Kilpatrick, Albert (‘Skip’) Rizzo y Josef Ruzek.

**Resultados**: Los panelistas debatieron estrategias innovadoras para la difusión del conocimiento científico y el tratamiento basado en la evidencia.

**Conclusiones**: Aunque existen tratamientos eficaces, es necesario mejorar la eficacia de estos tratamientos. También es necesario desarrollar estrategias más sencillas y de bajo costo para difundir los tratamientos eficaces. Sin embargo, los abordajes tecnológicos también ofrecen vías para una mayor difusión. Los investigadores deben comunicar los hallazgos científicos de un modo más efectivo para tener un impacto sobre la opinión pública y las políticas públicas.

## Abstract (zh)

**背景：**这篇文章基于创伤应激研究国际协会2016年11月在德克萨斯州达拉斯举办的第32届年度会议的一次小组会议。

**目的：**Paula Schnurr召集了一组在公共健康和技术领域的专家来讨论这个话题：‘我改变的想法和其中的原因’。

**方法：**讨论组里有Richard Bryant, Lucy Berliner, Dean Kilpatrick, Albert (‘Skip’) Rizzo和Josef Ruzek。

**结果：**讨论组成员关于科学知识传播和循证治疗的创新战略展开了讨论。

**结论：**尽管已经存在有效的治疗方法，仍然需要提高这些治疗方法的有效性，也需要发展更简单、低花费的策略来传播有效治疗。技术手段也提供了增加传播的途径。研究者们应该更有效地传播科学发现从而影响大众观点和公共政策。

Science, by its very nature, is a process of discovery that inevitably – if not always uniformly or quickly – leads to change in the understanding of natural and biological phenomena. The title of the book, *The Half-Life of Facts: Why Everything We Know Has An Expiration Date* (Arbesman, 2012), nicely captures this expectation of change and even argues that the ‘expiration’ of facts can be understood quantitatively.

The field of traumatic stress studies has undergone remarkable change, particularly since the formalization of the diagnostic criteria for posttraumatic stress disorder (PTSD) in 1980 (Schnurr, 2010). In 2015, a panel of experts in the neurobiology of PTSD who spoke at the annual meeting of the International Society for Traumatic Stress Studies (ISTSS) was asked to address the topic: ‘What I changed my mind about and why’ (Yehuda et al., 2016). For the ISTSS meeting in 2016, the organizers asked us to address this same topic from the perspectives of public health and technology, discussing what we have changed our mind about and the rationale for the change. Below we summarize our comments, in the order they were presented at the meeting.

## 1. Professor Dr. Paula Schnurr

My primary focus is in clinical trials of treatment for PTSD. My particular interest is in research to help us understand the effects of treatment in clinical practice – practical clinical trials of established treatments – and in promoting the use of evidence to guide policy and decision-making.

Since the first ISTSS Practice Guideline was published 16 years ago (Foa, Keane, & Friedman, 2000), we have learned even more convincingly about how to treat PTSD and other trauma-related problems. Simply put, treatment works. It works well for a range of people in a range of environments, and for a range of associated problems. We can offer real hope to trauma survivors around the world. We also can offer choice in the type of treatments and even in modalities for treatment.

What I have changed my mind about is how *well* treatments work. Please do not think that I am saying, ‘Treatments for PTSD don’t work.’ They do. The best treatments for PTSD meaningfully reduce PTSD and other comorbid symptoms and increase functioning and quality of life. What I am saying is that we have very effective treatments, but *we need to learn how to make more people more better*.

My colleague Dr. Juliette Harik has been leading an effort to develop an online decision aid for PTSD in order to enhance patients’ knowledge about treatment and support shared decision-making about treatment choice. We have been looking at loss of diagnosis following evidence-based treatment in order to optimally communicate information about treatment effectiveness. Loss of diagnosis is actually a very good way to measure clinically meaningful change; in one of my studies, it was associated with an average 40-point decrease on the Clinician Administered PTSD Scale and in achieving a good endpoint on measures of functioning and quality of life (Schnurr & Lunney, 2016). In the analyses performed to develop the decision aid (Harik, Grubbs, & Schnurr, 2016), we found that loss of diagnosis occurs for 53 out of every 100 patients who receive Prolonged Exposure (PE), Cognitive Processing Therapy, or Eye Movement Desensitization and Reprocessing, first-line psychotherapies recommended in practice guidelines (Forbes et al., 2010). For first-line medications, the selective serotonin reuptake inhibitors, the estimate was 42 out of 100. The effectiveness of these psychotherapies and medications is good, but why can’t it be better?

There have been critiques of the treatment literature that use numbers such as these to argue that PTSD treatments are ineffective and that we need new treatments. I disagree. We have so many good trauma-focused and non-trauma-focused treatments. There may be a role for novel approaches, but I think the primary way forward is to improve the treatments we have. We also need to study what to do for partial responders and non-responders. Do we add more sessions? Switch treatments? Add treatment for comorbid disorders? This is done routinely in clinical practice, but we really do not have an evidence base to guide us.

Some of the necessary research is ongoing. I think there should be more. PTSD is treatable, so we should do everything we understand how to help people fully recover.

## 2. Professor Dr. Richard Bryant

I have practised and researched as a clinical psychologist for over 25 years. In that time, I have focused my thoughts on achieving the best possible treatments for each patient I have treated – or in the case of research trials, strived for interventions that have attained the largest effect sizes. In doing this, I have worked in privileged settings where the health infrastructure, research support, qualifications of clinicians, and availability of sustained supervision for staff has never been a significant challenge to my goals.

And then I began undertaking projects in poorly resourced settings in Asia and Africa that had none of the resources that I had traditionally taken for granted. In these settings, primarily low- and middle-income countries, there are typically no mental health services (and often no substantive health infrastructure at all), few mental health specialists, and limited budgets to allocate to such services even in the wake of a major traumatic event. This is a worrying scenario because these settings are the most vulnerable to large-scale disasters, war and conflict, and interpersonal violence (Heise & Kotsadam, 2015).

As I have increasingly worked with World Health Organization (WHO) on public health initiatives, I have been increasingly convinced that our evidence-based interventions may need to be modified to be able to deliver useful strategies to settings where hundreds of thousands of people are in need but few mental health specialists exist. Although trauma-focused therapies have been shown to be efficacious in these settings (Morina, Malek, Nickerson, & Bryant, 2017), these approaches have rarely been scaled up because they require substantive training, lengthy duration of therapy, and sustained supervision. One public health approach is to try to implement evidence-based interventions that can be delivered to large numbers of affected people by non-specialist providers, even if it means that the effect size of treatment may not be as large as we would expect to see in a trauma-focused therapy delivered by expert specialists. Such an approach requires a shift in thinking about the expectations of (a) who delivers treatment, (b) length of treatment, (c) the minimum qualifications of the therapist, and (d) the length of training and supervision required. Recent meta-analysis suggests that this approach can be successfully implemented across a range of mental health conditions in low- and middle-income settings (Singla et al., 2017). For example, the WHO has developed a brief intervention that can be delivered by lay providers after reasonably brief training and supervision (Dawson et al., 2015). Although not trauma focused per se, initial evidence suggests it can reduce common mental health problems after trauma (Rahman et al., 2016).

This shift in my thinking does not involve throwing the baby out with the bathwater. Of course trauma-focused therapies are still my preference wherever resources permit. In fact, in some settings a stepped care framework may be appropriate where brief interventions may be offered to large numbers of affected people, and those who do not respond optimally to this approach may then be referred to health providers with capacity in more specialized trauma-focused therapy.

## 3. Professor Lucy Berliner

I used to believe, ‘if you build it they will come’. I thought offering training in effective trauma-specific treatments would be enough. That was based on my experience. We are a trauma specialty clinic in a university hospital. We began in the very early days of the field when there were no established trauma-specific treatments. As we became aware of treatments that might be effective we enthusiastically embraced training. This was long before the era of evidence-based treatment and the accumulation of effective treatments for trauma. We learned the new treatments as they came along.

Over time it became clear we could not serve all children and adults with trauma-specific impact in our community. About 10 years ago we embarked on an initiative to bring Trauma-Focused Cognitive-Behavioural Therapy (TF-CBT; Cohen, Mannarino, & Deblinger, 2016) to the public mental health system, where most children in our state receive mental health care. While some were interested to learn and apply the model, we encountered many unanticipated challenges to uptake of the treatment despite the evidence for its effectiveness and the free training.

Several lessons changed my perspective. First, the workforce typically is comprised of master’s-level clinicians with no common foundational training and little exposure to evidence-based psychotherapy (EBP). Principles and content that are central to EBPs are frequently unfamiliar. EBPs may be perceived as rigid or limiting autonomy. They are inconsistent with the eclectic, supportive, and non-specific approach that is usual care.

Second, like other EBPs, TF-CBT is harder work for both clinicians and clients because therapy is active and focuses on change. It is structured, focused, time limited, skill oriented, and measurement based. It also involves specific activities, including imaginal and in vivo exposure and cognitive processing, that are often novel and difficult skills for trainees to learn.

And third, from an organizational perspective, even when there is buy-in for EBPs, the challenges are enormous. Trauma-focused EBPs would only serve a minority of clients in general mental health settings. EBPs often address a single target, may be branded and require adherence to a specific model, and they typically require ongoing quality assurance. The dissemination and implementation literature assumes substantial and ongoing external funding to create organizational readiness, support delivery of the training, and maintain fidelity through monitoring for each EBP. These realities are the challenge for adoption of EBPs as standard practice.

In order to achieve the goal of widespread access to trauma-specific treatment and other EBPs, creative, low-cost methods will need to be in the mix with the externally supported approaches. Potential strategies include training methods that cover more than one EBP when based on the same theory and containing comparable components; transdiagnostic or common elements treatment approaches; train the trainer systems; cultivation and ongoing support for clinical supervisors as the primary sustainment mechanism; and practical methods for monitoring adherence, such as EBP structured progress notes. We will need to learn about and develop feasible approaches that are responsive to the real-world exigencies of those we are seeking to influence.

## 4. Professor Dr. Dean Kilpatrick

My first exposure to traumatic stress was as clinical psychology intern in 1968 attempting to understand and treat Vietnam veterans. My real involvement in the field began in 1974 as a founding member of South Carolina’s first rape crisis centre. I believed that research was the best way to identify and find effective treatments for rape-related mental health problems and that providing effective treatment would resolve most problems for most rape victims. My colleagues and I were among the first to conduct research on rape-related fear and anxiety, depression, and sexual dysfunction (e.g. Kilpatrick, Best, & Veronen, 1978; Kilpatrick, Veronen, & Resick, 1977, 1979). Through our experience with rape victims, we learned that stereotypes about rape and rape victims created numerous problems for victims who interact with the health care and criminal justice systems. We discovered that mental health, health care, criminal justice, and prevention resources for rape victims were inadequate and not a high priority.

I thought that using the scientific method to derive good research-based information was the key to solving all of these problems. Treatment providers would use interventions with the best empirical support. Systems would change once they got accurate information about what was needed. Policy makers would change laws and provide adequate resources once they had accurate data. Everyone would quit blaming rape victims once they got sound information. We got more accurate information, but none of these things actually happened.

In my mind, research is still critically important, especially treatment outcome and implementation research. However, I now know four things. First, the scientific method is under attack, and most people and policy makers neither understand nor value science. Therefore, research findings have less impact on public opinion, public policy, and clinical practice than I expected. This is partially because researchers do a poor job of communicating the real-life implications and value of research findings. Second, many problems and needs of victims are not fully addressed by even the best mental health treatment, so we need public health perspectives, approaches, and interventions (Magruder, Kassam-Adams, Thoresen, & Olff, 2016). This includes using public health multi-media strategies to communicate research-informed prevention and intervention messages about trauma in a user-friendly way.

Third, I know now that influencing public policy is essential to get the laws, regulations, policies, and funds needed to help those we serve (e.g. Kilpatrick & Ross, 2001). Effective communication of research findings can impact public policy through public education (e.g. Kilpatrick, Edmunds, & Seymour, 1992), so we should do more of this. Fourth, I underestimated how hard it would be and how long it would take to address these problems. Change is hard and takes a really long time. We remain far from where we should be, but we must keep plugging away because trauma victims deserve nothing less than our full effort for as long as it takes.

## 5. Professor Dr. Skip Rizzo

Over the last 20 years, my lab has focused on developing and testing virtual reality (VR) systems (Rizzo et al., 2013). The guiding principle underlying this effort is that VR is a tool for amplifying the impact of evidence-based cognitive-behavioural treatment and extending the skills of already well-trained clinicians – not replacing them! This principle was highlighted in our efforts to deliver PE using VR exposure (VRE). The core assumption was that VR technology supports the creation of customized, relevant, and immersive virtual experiences that help PTSD clients to confront and process difficult trauma-related emotional memories within the safety of the clinical setting. VRE is also hypothesized to reduce the reliance on the hidden world of imagination to promote client engagement with their trauma narrative, and thus circumvent the hallmark PTSD symptom of avoidance.

I always believed that PE was enough to produce positive clinical outcomes; adding additional treatment components into the mix often delivered little incremental value. More recently, I have come to believe we need a more comprehensive approach to address the behavioural health challenges faced by the current generation of military trauma survivors. I have become more accepting of the need for more varied, stepwise, and multi-component approaches to treating the variety of ill effects that can occur with traumatic exposure. From this, I have come to think that we need to spend more time offering a variety of treatment options to patients, some in the complementary and alternative medicine domain that, while not rock-solid in terms of multiple independent randomized clinical trials, may connect individuals to the concept of healing.

I see the first challenge being in breaking down barriers to care for getting someone into some form of treatment – any form to start – but with the stated plan that this is just one step in a longer journey that will be available to the client on a path toward wellness. I further believe that the common barriers to care require as much focus as we put into studying treatment process and efficacy. More effort is needed to build trauma survivors’ awareness of the range of available treatment options, increase the perception of anticipated benefits, make treatment more accessible and make available more well-trained treatment providers, and finally, make help-seeking more acceptable (reducing stigma), all in the service of promoting adherence to an often difficult and painful road to health. PE or other evidence-based treatment can be the final step on the road if a survivor cannot be persuaded or is not ready to engage in a trauma-focused approach initially.

Accomplishing these ends may require clinicians to consider the use of technology options beyond VRE, teletherapy, and mobile apps to amplify the effects of known treatment processes and break down barriers to care. Perhaps clinicians should take note of the advances in the use of artificially intelligent virtual human support guides that can engage clients on their computer monitor or mobile phone screen. These technology approaches, while in their infancy now, need to be studied as new options for breaking down barriers to care and creating more customized and comprehensive paths to healing and achieving growth.

## 6. Professor Dr. Joe Ruzek

As an educator, I always assumed that delivery of effective training represented a primary and perhaps the most important strategy for improving care of trauma survivors. For 15 years, I oversaw a 30-hour PTSD Clinical Training Programme attended by over 2500 mental health clinicians and much lauded by participants. More recently, I helped lead a national training programme in PE treatment for PTSD in the U.S. Department of Veterans Affairs. PE training was effective in winning the hearts and minds of clinicians, improving their skills, and achieving large reductions in PTSD symptoms among Veterans treated during the training process. This state-of-the-art training combined a 4-day training workshop with weekly telephone consultation for the duration of two cases. The training worked, but it was expensive to gather clinicians for face-to-face training, and post-training consultation proved difficult to implement. Such training could be offered very occasionally for major priorities, but not as a means of addressing the large body of existing and emerging training needs. And we found that skills learned during training were not necessarily then implemented in routine care.

What about combining online training plus telephone supervision as an alternative? I conducted two randomized clinical trials on this subject, but encountered difficulties with clinician recruitment and attendance, and the measurement of skills improvement among those trained presented enormous challenges. More generally, there are no standing resources to implement widespread training, consultation, and implementation in most organizations or communities. And anyway, training focuses largely on individual treatment, which requires relatively intensive staffing resources that will seldom be available; even if trained, individual treatment providers cannot adequately serve the large populations of individuals needing their services.

But if training individual practitioners is not an adequate solution to the challenges of treatment improvement, what is? This quandary started me thinking about Internet and phone-based interventions. If we can place elements of interventions on these technologies, perhaps we can get excellent treatment with a much-reduced training burden. Technology might enable the free delivery of best practices on reliable basis, improve the impact of client self-care, enable peers and paraprofessionals to offer more to their clients, and help clinicians become more effective and address more of the problems faced by their clients. Technologies might support delivery of evidence-based treatments, and accelerate movement towards measurement-based care by facilitating outcomes monitoring and data gathering. I now believe that phone and web technologies, if systematically developed, evaluated, and thoughtfully integrated with in-person care, will transform mental health practices and enable development of a 21st century stepped care mental health delivery system.

## 7. Summary

A central theme underlying all of these presentations is that the field of traumatic stress studies has developed a range of effective treatments for individuals who are exposed to traumatic events. Yes, as Schnurr argued, there is a need to enhance the effectiveness of these treatments, but the message is clear: treatment works. However, the presentations also emphasized additional messages about the need for dissemination and innovation, along with the use of technology, in order to achieve optimal reach. In addition, researchers must communicate scientific findings more effectively to impact public opinion and public policy.

Trauma is a global problem (Schnyder, 2013). Simple but effective strategies are needed to enable local providers in countries that have limited mental health resources to effectively treat trauma-related disorders. In order to achieve the goal of widespread access to trauma-specific treatment and other EBPs, creative, low-cost methods will need to be in the mix with the empirically supported approaches. The innovations may be low tech, but technology approaches need to be studied as new options for breaking down barriers to care and creating more customized and comprehensive paths to healing and achieving growth. Phone and web technologies have the potential to transform mental health practices and enable development of a 21st century stepped care mental health delivery system.

## Highlights of the Article

- There is a need to enhance the effectiveness of even the most effective treatments for trauma-related disorders.
- We need to develop creative, low-cost strategies in order to achieve widespread access to these treatments.
- Technology approaches need to be studied as new options for breaking down barriers to care and enabling development of a 21st century mental health delivery system.
- Researchers must communicate scientific findings more effectively to impact public opinion and public policy.

## References

[CIT0001] Arbesman S. (2012). *The half-life of facts: Why everything we know has an expiration date*. New York, NY: Penguin.

[CIT0002] Cohen J., Mannarino A., & Deblinger E. (2016). *Treating trauma and traumatic grief in children and adolescents* (2nd ed.). New York, NY: Guilford.

[CIT0003] Dawson K. S., Bryant R. A., Harper M., Kuowei Tay A., Rahman A., Schafer A., & van Ommeren M. (2015). Problem Management Plus (PM+): A WHO transdiagnostic psychological intervention for common mental health problems. *World Psychiatry*, 14, 354–6. doi:10.1002/wps.20255 26407793 PMC4592660

[CIT0004] Foa E. B., Keane T. M., & Friedman M. J. (Eds.). (2000). *Effective treatments for PTSD: Practice guidelines from the International Society for Traumatic Stress Studies*. New York, NY: Guilford.

[CIT0005] Forbes D., Creamer M., Bisson J. I., Cohen J. A., Crow B. E., Foa E. B., … Ursano R. J. (2010). A guide to guidelines for the treatment of PTSD and related conditions. *Journal of Traumatic Stress*, 23, 537–552. doi:10.1002/jts.20565 20839310

[CIT0006] Harik J. M., Grubbs K. G., & Schnurr P. P. (2016, 11). Using graphics to communicate information about PTSD treatment effectiveness to patients In Hamblen J. L. (Chair). *Enhancing the quality of online information to support PTSD treatment engagement. Symposium presented at the 32nd annual meeting of the International Society for Traumatic Stress Studies*, Dallas, TX.

[CIT0007] Heise L. L., & Kotsadam A. (2015). Cross-national and multilevel correlates of partner violence: An analysis of data from population-based surveys. *Lancet Global Health*, 3, e332–340. doi:10.1016/S2214-109X(15)00013-3 26001577

[CIT0008] Kilpatrick D. G., Best C. L., & Veronen L. J. (1978). The adolescent rape victim: Psychological responses to sexual assault and treatment approaches In Kreutner A. & Hollingsworth D. (Eds.), *Adolescent obstetrics and gynecology* (pp. 337–359). Chicago, IL: Year Book Medical Publishers.

[CIT0009] Kilpatrick D. G., Edmunds C., & Seymour A. (1992). *Rape in America: A report to the nation*. Arlington, VA: National Victim Center and the Crime Victims Research and Treatment Center, Charleston, SC.

[CIT0010] Kilpatrick D. G., & Ross M. E. (2001). Torture and human rights violations: Public policy and the law In Keane T., Gerrity E., & Tuma F. (Eds.), *Mental health consequences of torture* (pp. 317–331). New York, NY: Kluwer Publishing.

[CIT0011] Kilpatrick D. G., Veronen L. J., & Resick P. A. (1977). Responses to rape: Behavioral perspectives and treatment approaches. International Congress of Behaviour Therapy, Uppsala, Sweden. *Scandinavian Journal of Behavior Therapy*, 6(SUPPL 4), 1977.

[CIT0012] Kilpatrick D. G., Veronen L. J., & Resick P. A. (1979). The aftermath of rape: Recent empirical findings. *American Journal of Orthopsychiatry*, 49, 658–669. doi:10.1111/j.1939-0025.1979.tb02651.x 495705

[CIT0013] Magruder K. M., Kassam-Adams N., Thoresen S., & Olff M. (2016). Prevention and public health approaches to trauma and traumatic stress: A rationale and a call to action. *European Journal of Psychotraumatology*, 7, 29715. doi:10.3402/ejpt.v7.29715 26996536 PMC4800286

[CIT0014] Morina N., Malek M., Nickerson A., & Bryant R. A. (2017). Meta-analysis of interventions for posttraumatic stress disorder and depression in adult survivors of mass violence in low- and middle-income countries. *Depression and Anxiety*, 34, 679 –691. doi:10.1002/da.22618 28419625

[CIT0015] Rahman A., Hamdani S. U., Riaz N., Bryant R. A., Dawson K., Firaz M., … van Ommeren M. (2016). Effect of a psychological intervention using problem-solving and behavioral therapy on anxiety and depressive symptoms in adults with psychological distress in conflict-affected Pakistan: A randomized clinical trial. *J* *AMA*, 316, 2609–2617. doi:10.1001/jama.2016.17165

[CIT0016] Rizzo A. A., Buckwalter J. G., Forbell E., Reist C., Difede J., Rothbaum B. O., … Talbot B. (2013). Virtual reality applications to address the wounds of war. *Psychiatric Annals*, 43, 123–138. doi:10.3928/00485713-20130306-08

[CIT0017] Schnurr P. P. (2010). PTSD 30 years on. *Journal of Traumatic Stress*, 23, 1–2. doi:10.1002/jts.20498 20131321

[CIT0018] Schnurr P. P., & Lunney C. A. (2016). Symptom benchmarks of improved quality of life in PTSD. *Depression and Anxiety*, 33, 247–255. doi:10.1002/da.22477 26882293

[CIT0019] Schnyder U. (2013). Trauma is a global issue. *European Journal of Psychotraumatology*, 4, 20419. doi:10.3402/ejpt.v4i0.20419 PMC3589434 23469313

[CIT0020] Singla D. R., Kohrt B., Murray L. K., Anand A., Chorpita B. F., & Patel V (2017). Psychological treatments for the world: Lessons from low- and middle-income countries. *Annual Review of Clinical Psychology*, 13, 149 –181. doi:10.1146/annurev-clinpsy-032816-045217 PMC5506549 28482687

[CIT0021] Yehuda R., Spiegel D., Southwick S., Davis L. L., Neylan T. C., & Krystal J. H. (2016). What I have changed my mind about and why. *European Journal of Psychotraumatology*, 7, 33768. doi:10.3402/ejpt.v7.33768 27837585 PMC5106864

